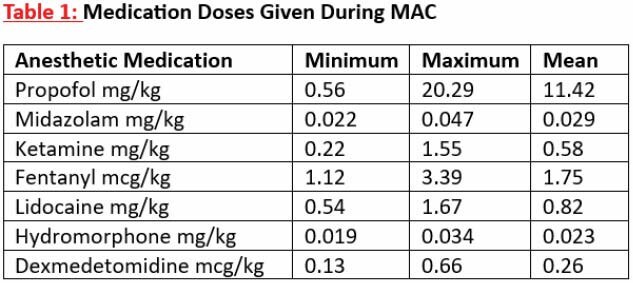# 852 Safety and Efficacy of Multiple Monitored Anesthesia Care During Burn Dressing Change: Three Case Series

**DOI:** 10.1093/jbcr/iraf019.383

**Published:** 2025-04-01

**Authors:** Fatma Ulusan Sayali, Dhaval Bhavsar, Duncan Nickerson, Julia Slater, Katherine Golson, Niaman Nazir, Anthony Kovac

**Affiliations:** The University of Kansas Health System, Burnett Burn Center; The University of Kansas Health System, Burnett Burn Center; The University of Kansas Health System, Burnett Burn Center; The University of Kansas Health System, Burnett Burn Center; The University of Kansas Health System, Burnett Burn Center; The University of Kansas Health System, Burnett Burn Center; The University of Kansas Health System, Burnett Burn Center

## Abstract

**Introduction:**

Monitored Anesthesia Care (MAC) is a technique that enables a smooth workflow of patient care during burn dressing change (BDC) with concurrent satisfaction ratings among patients, burn nurse/techs and anesthesia providers. While the safety and efficacy of MAC has previously been described, safety and efficacy of multiple MACs for BDC has not been reported previously.

**Methods:**

This prospective observational study, approved by our hospital IRB, was conducted from October 7, 2023, to March 31, 2024. Three male burn patients who underwent multiple (13,16 and 22 each) BDCs under MAC were analyzed. To evaluate the safety of MAC, the duration, anesthetic choice, order and instances of hypoxia, apnea or hypotension were noted. Mean arterial pressure (MAP) changes and pain scores were evaluated to look for any correlation with medication choice or pain outcome reported by the patient.

**Results:**

Individual MAC durations varied from 45 to 150 minutes. Propofol was used in all cases, followed in descending order of midazolam, ketamine, and fentanyl. The number of medications varied from 4 to 7 in each MAC. Incidence of apnea, hypoxia, and hypotension was 37, 6 and 36, respectively. Each instance was recognized and treated properly without any ill effects using interventions such as jaw thrust, repositioning or administering vasopressors. Anesthetic medication dosages are shown in Table 1. After multiple MACs, no lab deterioration or tachyphylaxis was observed. The difference between the start and end MAP values were within the < 20 mmHg range in 48 cases. More fluctuations in MAP values were seen when the patients were under anesthesia. Comparing pre- vs post-MAC pain scores showed an increase in 14 cases, no change in 15, and decrease in 22.

**Conclusions:**

Although there is not a standardized approach regarding MAC for BDC, the multiple medication variety administered did not appear to affect the safety and efficacy of the three patients who received multiple MACs. However, more studies are needed to recommend combinations of medications and dosages to achieve more stable hemodynamics and better analgesia both during and after MAC.

**Applicability of Research to Practice:**

Administration of multiple MAC anesthetics for burn dressing change is safe and effective with no laboratory deterioration or medication tachyphylaxis.

**Funding for the Study:**

N/A